# SB Digestor: a tailored driver gene identification tool for dissecting heterogeneous Sleeping Beauty transposon-induced tumors

**DOI:** 10.7150/ijbs.81317

**Published:** 2023-03-13

**Authors:** Aiping Zhang, Lijian Wang, Josh Haipeng Lei, Zhengqiang Miao, Monica Vishnu Valecha, Peng Hu, Kai Miao, Chu-Xia Deng

**Affiliations:** 1 Cancer Center, Faculty of Health Sciences, University of Macau, Macau SAR, China.; 2 Centre for Precision Medicine Research and Training, Faculty of Health Sciences, University of Macau, Macau SAR, China.; 3 Genomics & Bioinformatics Core, Faculty of Health Sciences, University of Macau, Macau SAR, China.; 4 College of Fisheries and Life Science, Shanghai Ocean University, Shanghai, China.; 5 MoE Frontiers Science Center for Precision Oncology, University of Macau, Macau SAR, China.

**Keywords:** Sleeping Beauty transposon, intertumor heterogeneity, common insertion sites, SB Digestor, Fgfr2

## Abstract

Sleeping Beauty (SB) insertional mutagenesis has been widely used for genome-wide functional screening in mouse models of human cancers, however, intertumor heterogeneity can be a major obstacle in identifying common insertion sites (CISs). Although previous algorithms have been successful in defining some CISs, they also miss CISs in certa*in situ*ations. A major common characteristic of these previous methods is that they do not take tumor heterogeneity into account. However, intertumoral heterogeneity directly influences the sequence read number for different tumor samples and then affects CIS identification. To precisely detect and define cancer driver genes, we developed SB Digestor, a computational algorithm that overcomes biological heterogeneity to identify more potential driver genes. Specifically, we define the relationship between the sequenced read number and putative gene number to deduce the depth cutoff for each tumor, which can reduce tumor complexity and precisely reflect intertumoral heterogeneity. Using this new tool, we re-analyzed our previously published SB-based screening dataset and identified many additional potent drivers involved in Brca1-related tumorigenesis, including Arhgap42, Tcf12, and Fgfr2. SB Digestor not only greatly enhances our ability to identify and prioritize cancer drivers from SB tumors but also substantially deepens our understanding of the intrinsic genetic basis of cancer.

## Introduction

The Sleeping Beauty (SB) DNA transposon system is a reconstructed Tc1-like transposon that is derived from fish 1. This system consists of a conditionally expressed transposase and mutagenic transposon allele, which is flanked by inverted repeats/direct repeats 2, 3. The transposase directs the transposon cut-and-paste mechanism by catalyzing the excision from its original location and promoting its reintegration into TA dinucleotides elsewhere in the genome. Because of this unique characteristic of SB, it is able to truncate tumor suppressors and/or activate oncogenes simultaneously in spontaneously developed tumors in the mouse model, which more closely mimics conditions for human tumor initiation and development. Thus, sequencing transposon insertion sites from tumor samples enable driver gene identification and reveal cancer-related pathways, which provide insight into the mechanisms underlying cancers. 4. So far, the SB transposon has been used to identify driver genes in multiple types of cancers, including breast cancer 5, melanoma 6, osteosarcoma 7, liver cancer 8, pancreatic cancer 9, colorectal cancer 10, nervous system cancer 11 and other tumors 2. Further interrogation of the SB-tagged mutations could facilitate the identification of the sophisticated drivers that are responsible for several important aspects of cancer, including tumorigenesis, metastasis 12, 13, tumor microenvironment influences 14, and *in vivo* drug resistance 15.

To distinguish the genes involved in promoting tumorigenesis, high-throughput DNA sequencing was performed for SB-driven tumors to identify the transposon-activated and transposon-trapped genes. Several statistical algorithms have been developed to determine the hot spots of SB insertion loci in the tumor genome, including TAPDANCE 16, and SB Driver Analysis 17, Gaussian kernel convolution (GKC) 18, and gene-centric common insertion sites (gCISs) 19. These approaches successfully defined some driver genes in previous studies. For example, locus-centric algorithms such as TAPDANCE and GKC can effectively identify highly condensed SB insertion sites but are moderately effective in defining scattered SB insertions. To solve this problem, Newberg et al. developed SB Driver software to identify tag mutations in an unbiased manner 17. Nevertheless, the sequence depth cutoff determination of this software was experience-based, which could compromise its ability to eliminate artificial effects and correctly account for the tumor diversity.

Tumor heterogeneity describes the observation that different tumor cells demonstrate distinct phenotypic and genotypic profiles, including distinct cellular morphology, gene expression, and driver genes 20. Similarly, the number and the type of driver genes of SB tumors exhibit major differences, as revealed by our previous study carried out by analyzing 306 Brca1-related tumors using TAPDANCE 5. Therefore, analysis relying on a uniform depth cutoff for all tumor samples would lead to either the inclusion of some background noise or the elimination of some potent drivers. Specifically, based on the calculation principle, when we choose an unsupervised and uniformed depth cutoff in a given tumor, the number of sequenced reads will greatly affect the identification of driver genes, i.e., more reads will generate a greater number of driver genes and vice versa. Moreover, the landscape of intertumor heterogeneity is reflected by striking molecular and biological variations. Thus, to better assess the natural course of the tumor, it is required to truly individualize read depth cutoff for the driver gene analysis of different tumors.

Therefore, in this study, we developed the SB Digestor, which is a tailored SB driver gene identification approach that could initially distinguish a variety of driver genes for individual tumors based on saturation analysis of the putative drivers. This tool helped us to elicit the intertumor heterogeneity effect and then diagnose driver genes for SB tumors with high precision. To illustrate the power of SB Digestor, we used it to reanalyze data from Brca1-related tumors (n=306) and identified 170 candidate cancer driver genes, including 121 potential drivers that were not identified by our earlier study 5. The newly identified genes included several well-known cancer drivers, such as Fgfr2, Hras, Tgfbr2, Nf1, and Erbb2, as well as others whose function in cancer remains elusive. Finally, we conducted functional validation using Fgfr2, Arhgap42, and Tcf12 to illustrate their roles in BRCA1-associated tumorigenesis.

## Results

### The overall design of the SB Digestor

**Figure 1:**
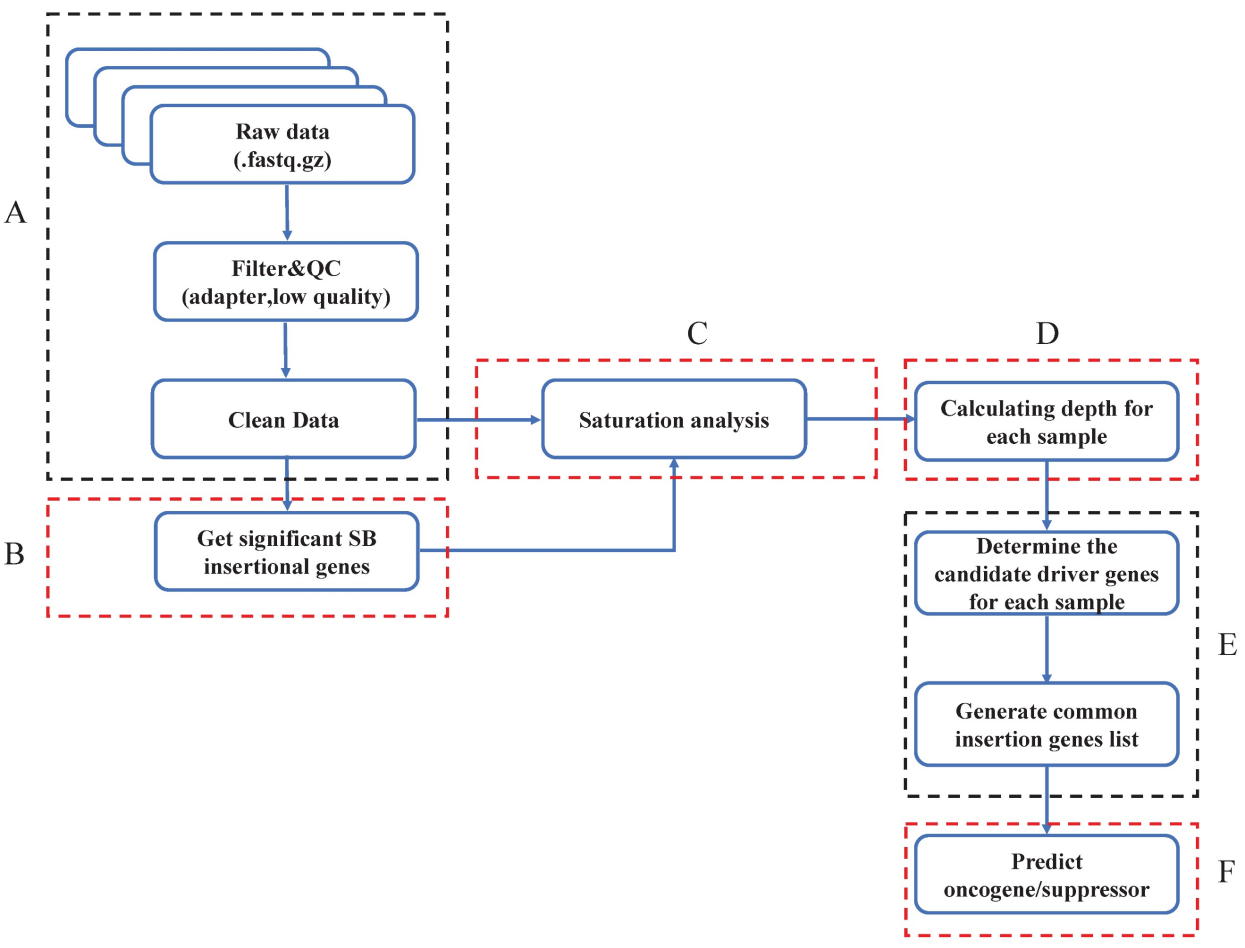
** Overview of SB Digestor analysis pipeline**. **A.** Raw data pre-processing. The raw data were processed by filtering the low-quality reads and trimming the adapters. **B**. Define significant insertional genes by binomial test.** C.** Saturation analysis. To determine the sequencing depth cutoff, 50 sample sizes of reads were extracted randomly, followed by gene annotation. Then, a curve was fitted, and an adapted formula was obtained to reflect the correlation between the number of annotated genes and the 50 sample sizes of reads for each sample. **D.** Defining depth for each sample. The depth cutoff value for each sample was calculated with the formula depth=reads num/gene number. **E.** Identify drivers. The candidate driver genes for each sample were sorted out based on the depth cutoff. Then, generate common insertion genes list for all tumors. **F.** Characterize drivers. The driver genes were further characterized based on the SB transposon insertion patterns, including both locations and transposon promoter directions.

The SB Digestor includes 6 modules, which are critical for the unbiased identification of cancer drivers. After obtaining the SB cancer datasets, we filtered the low-quality of reads and trimmed the adapters (Fig. 1A). In order to enhance the ability to analyze and interpret cancer drivers, we gave sufficient consideration to one of the most important tumor biology features-the tumor heterogeneity. We try to design a strategy to individualize depth cut-off for each sample to replace the previous tools used-the uniform and empirical depth values. So, with clean data, we'll first get significant SB insertional genes with the binomial test and generate a gene library for each sample (Fig. 1B). Later, saturation analysis would be executed to depict the relationship between the read number and the significant SB insertional gene number (Fig. 1C). Based on the above relationship, the depth cutoff calculation formula could be deduced (Fig. 1D). Then, we can determine the candidate drive genes for each sample and generate the common insertion gene list of all tumors (Fig. 1E). Finally, we predicted and characterized whether a candidate driver gene is an oncogene or a tumor suppressor (Fig. 1F).

#### Get significant SB insertional genes within the whole genome

**Figure 2:**
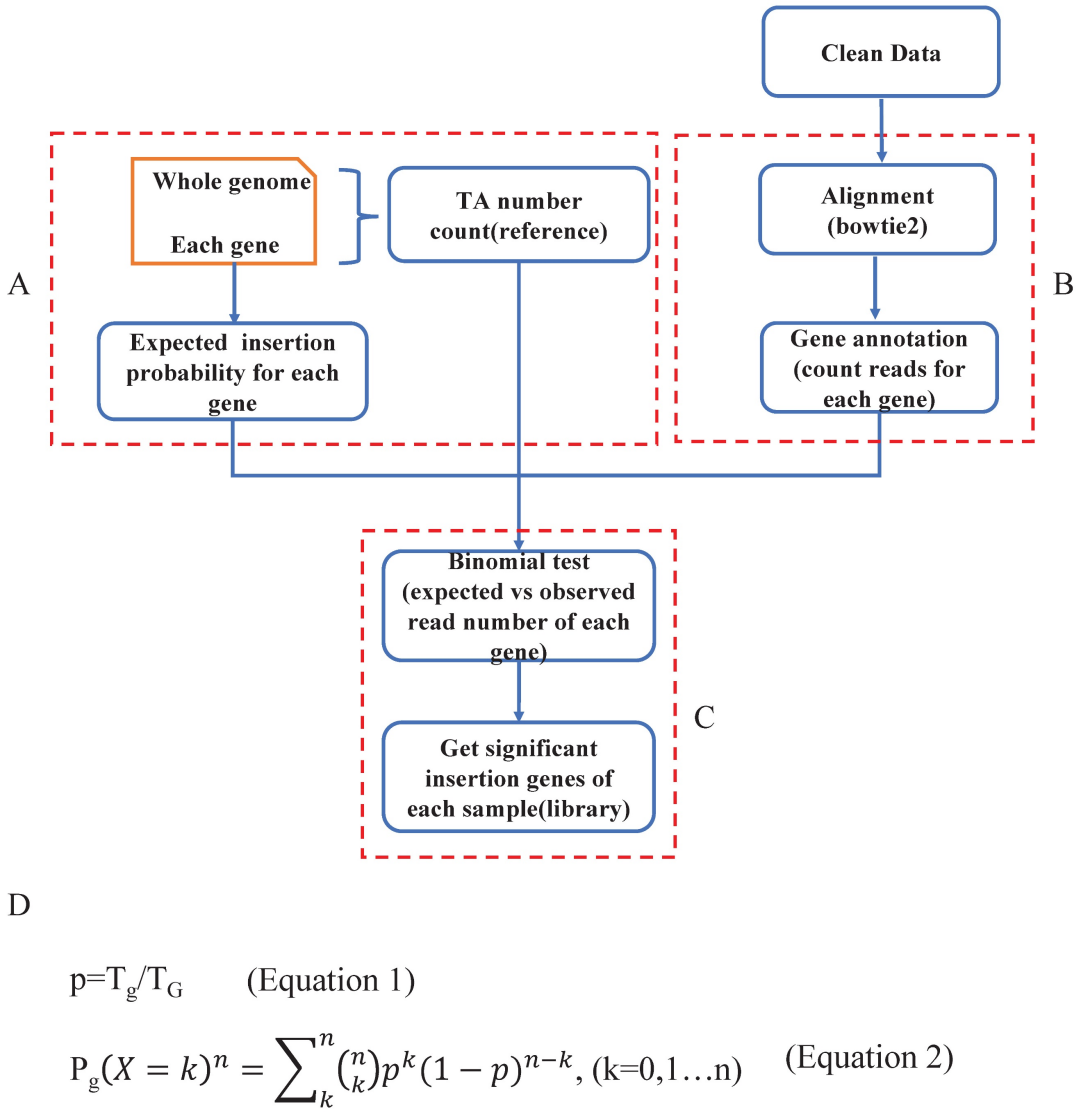
** Define significant SB insertional genes. A. Calculate the expected SB insertion probability of each gene.** The expected trapping probability of each gene in the mouse genome was calculated based on the gene size and the number of TA dinucleotides. **B.** Clean data alignment and annotation. After the data pre-processing, the clean data was mapped to the mouse reference genome, and did the loci annotation. **C.** The binomial test was applied to sort the significant SB insertional genes for each sample and generate a gene library for each sample. **D.** The equations to calculate the expected SB insertion probability (Equation 1) and the binomial P value of each gene (Equation 2), where Tg is the number of TA sites in a given gene and T_G_ is the number of TA dinucleotides in the whole genome. p is the probability of a transposon jumping into the given gene within the whole mouse genome (Equation 1); k is the observed insertion number in a certain gene, which is also the mapped read number of the gene. P_g_ is the binomial probability (Equation 2).

Previous studies have indicated that Sleeping Beauty transposons insert only into TA dinucleotides. To identify genes with significant SB transposon insertions, we first calculated the expected insertion probability for each gene (Fig. 2A). Specifically, we counted the TA (T_G_) dinucleotides for the whole mouse reference genome (mm10), and the number was 88475427. Then, we calculated the number of TA dinucleotides in each gene (T_g_) (Supplementary Table 1). To detect oncogenic insertions, we extended the TA counting region to 3000 bp upstream of each gene. Then we did the data mapping and gene annotation, counting reads for each gene of each sample (Fig. 2B). Since Sleeping Beauty transposons randomly jump within the mouse genome, the probability of SB insertion in each TA dinucleotide is the same (1/T_G_). Additionally, each SB insertion is independent, which means that none of the insertions affect the probability of other insertions. Thus, we assumed that our distribution is binomial (Fig. 2C). To test whether a given observed insertion number of a certain gene is a significant insertion, we calculated the insertion probability of each gene p by Tg/T_G_ (Fig. 2D-Equation 1) and its binominal P value by using the formula (Fig. 2D-Equation 2). If a gene had a P_g_ smaller than 0.05, we defined it as a significant SB insertion gene. Then, we evaluated the statistical significance of each SB insertion gene and generated a gene library for each sample.

#### Determination of read depth cutoff for each sample with saturation analysis

**Figure 3:**
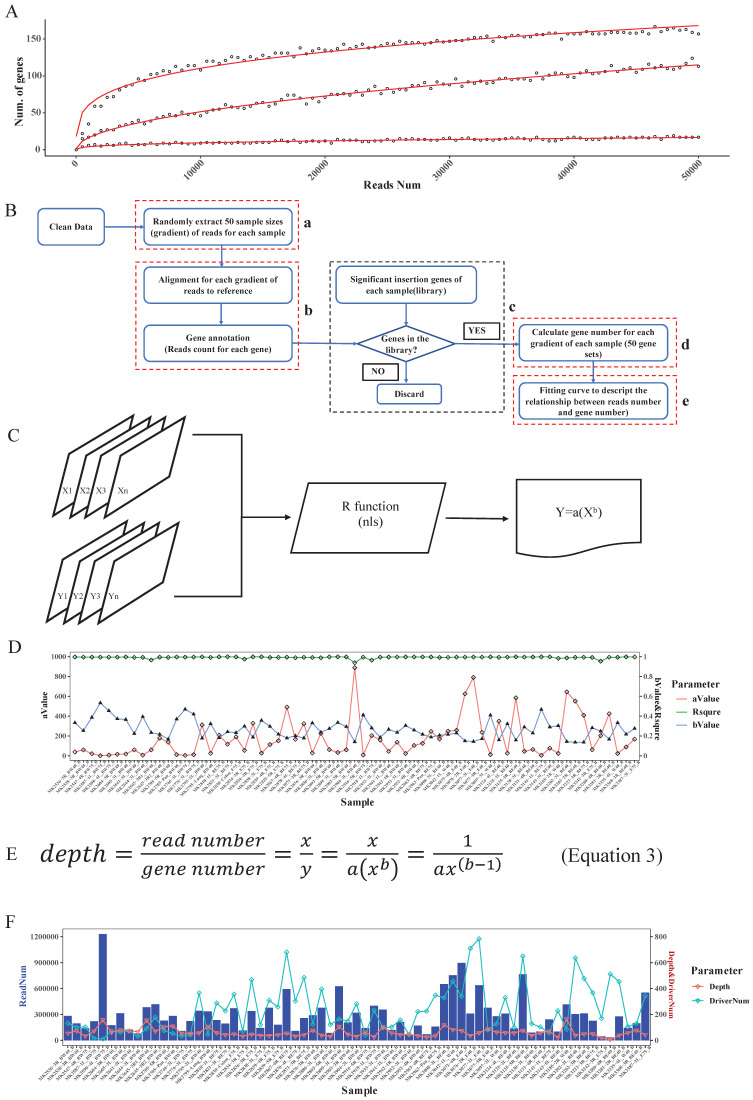
** Read depth determination**. **A.** Correlation curve of input read number and annotated significant insertional gene number. Here, the three curves represent 3 different tumor samples. For each sample, we extracted the same number of reads and then mapped and annotated them one by one. For a certain sample, if an annotated gene exists in the previous binomial test statistics library, we deemed it to be a reliable insertional gene. **B.** The strategy of saturation analysis. B-a. For each sample, 50 sample sizes of reads were randomly extracted. B-b. Alignment and gene annotation were applied for each sample size of reads, then counted the number of reads number for each gene. B-c.d. Statistical the significant SB insertional gene number and generate 50 gene sets for each sample. B-e. Fitting a curve to descript the relationship between reads number and gene number by the 50 sample sizes of reads and the corresponding gene sets. **C.** Flowchart of fitting curve calculation. We used the R function nls to deduce the relationship between the read number and gene number (X: 50 sample sizes of read number, Y: corresponding gene number, both a and b are constant). **D.** The a, b, and R squared values of each sample. **E.** The depth calculation formula, where y is the total significant insertion gene number of each sample; and x is the total clean read number of each sample. **F.** The read number, the calculated depth cutoff, and the detected candidate driver gene numbers of 67 test samples.

Based on the driver gene analysis principle in a previous study, more candidate driver genes could be identified with more sequenced reads 19. We randomly selected 3 SB tumor samples and annotated the genome locations, we indeed found that using a greater number of reads for a particular tumor increased the capacity of identifying the putative genes (Fig. 3A). Moreover, due to tumor heterogeneity, the number and the type of cancer driver genes in different tumors could vary 21. In our test samples, as the figure shows, that different tumors have different numbers of putative driver genes (Fig. 3A).

In addition, when calling tumor driver genes with SB screening tumors, due to the diversity of sequencing coverage in different genes, a read depth cut-off was usually applied. However, this cut-off value was empirically determined. If an unsupervised depth cutoff is set for all tumor samples, as was done in previously reported tools, the number of detectable putative driver genes is different under different depth cutoffs. For example, if we have 3 tumors with the same number of sequenced reads, 10000, due to tumor heterogeneity, if the driver gene numbers of these 3 tumors are 10, 100, 1000, then the average depths of these genes are 1000, 100, 10. Therefore, if we set 1000 as the depth cutoff, we would miss many true-positive candidate genes in the last two samples, but if we set it as 10, we may obtain many false-positive candidate genes in the first two samples. Therefore, this approach is not conducive to accurately finding true SB insertion genes. Also, different samples showed different slopes, which means that different samples require different read numbers to detect the same number of candidate genes (Fig. 3A). Thus, it is not reasonable to uniform the depth cutoff to all tumor samples when calling the SB insertion genes, rather, this value should be tailored for different tumor samples.

To address the above problems, in our algorithm, we not only consider the statistical significance like other tools did but the biological aspect (tumor heterogeneity) was also taken into account to identify tumor driver genes. First, for each sample, we computed the clean read number. Next, we randomly extracted 50 sample size numbers (gradients) of reads for each sample to conduct the saturation analysis. For example, if a sample had 10000 reads, the gradient is 200, and the extracted sample sizes were 200, 400, 600, 800, 1000, 1200…10000 (Fig. 3B-a). Here, for each read sample size, we did the alignment and annotated the reads to obtain the located genes (Fig. 3B-b), and once the annotated gene existed in the previous significant insertional gene library of the sample, we took it into account and calculated the gene number for each sample size (Fig. 3B-c). Thus, for these 50 sample size numbers of reads of each sample, we obtained 50 gene sets (Fig. 3B-d). Following that we plotted the correlation curve to depict the read number and candidate gene number, which is called saturation analysis. (Fig. 3B-e). Then, we used the R function “nls” (Nonlinear Least Squares) to estimate the parameters a and b of this nonlinear model (Fig. 3C) to digitalize the relationship between read number and gene number. To evaluate the accuracy of the fitting curve, we estimated the R-squared, which is widely used to evaluate how well the data fit the regression model. Then, we tested the model with DNA sequencing data from 67 Brca1^co/co^ SB tumor samples. The R-squared value of these samples showed that the formula we established here could exactly reflect the correlation between read number and gene number with particular “a” and “b” values for each tumor sample (Fig. 3D).

Based on the fitting curve formula, we were able to calculate the sequencing depth (Fig. 3E) for each individual sample under different sequenced read numbers. For each test sample, we obtained a depth (Fig. 3F), and we set this depth as the depth cutoff for each sample. For example, the depth we calculated for sample 'MK3242-3R_E75_B' was 169, and the significant insertion number (determined by the binomial test, and P value=0) of Fgfr2 in this sample is 513. Thus, we defined Fgfr2 as a putative candidate gene for this sample.

In the test samples, as we expected, the intertumoral heterogeneity was reflected in the number of putative driver genes. We might obtain tons of sequenced reads in an SB tumor, but after analysis, we only got a few of the putative driver genes. For example, a sample MK2590-4L_BW75_B had the most reads (Fig. 3F), but more than 97% of them were located at position 100383957 of chromosome 11, within the gene 'Jup' region. Finally, only 11 candidate genes were identified in this sample. Inversely, sample MK3280-4R_BE40_B got more than 500 candidate drivers with less than a total of 34411 reads. Thus, these results indicate that an individualized depth cutoff is necessary for SB tumor driver gene identification analysis.

#### Determination of common insertion genes and classification of oncogenes and tumor suppressors

To call common insertion genes among the above candidate genes across all tumors, we used an appearance in at least 3 or more than 5% of all tumors as a cutoff. To estimate whether the candidate gene is an oncogene or a tumor suppressor, we evaluated the insertion patterns of transposons in each candidate gene (i.e., whether the insertions are clustered within a hotspot region or widely distributed and in the same or opposite direction as transcription of the host gene). If more than 50% were forward insertion reads and only a few hotspot insertion regions were found, we defined the gene as an oncogene; otherwise, the host gene was presumed to be a tumor suppressor.

### Comparison of SB Digestor with TAPDANCE and SB Driver

**Figure 4:**
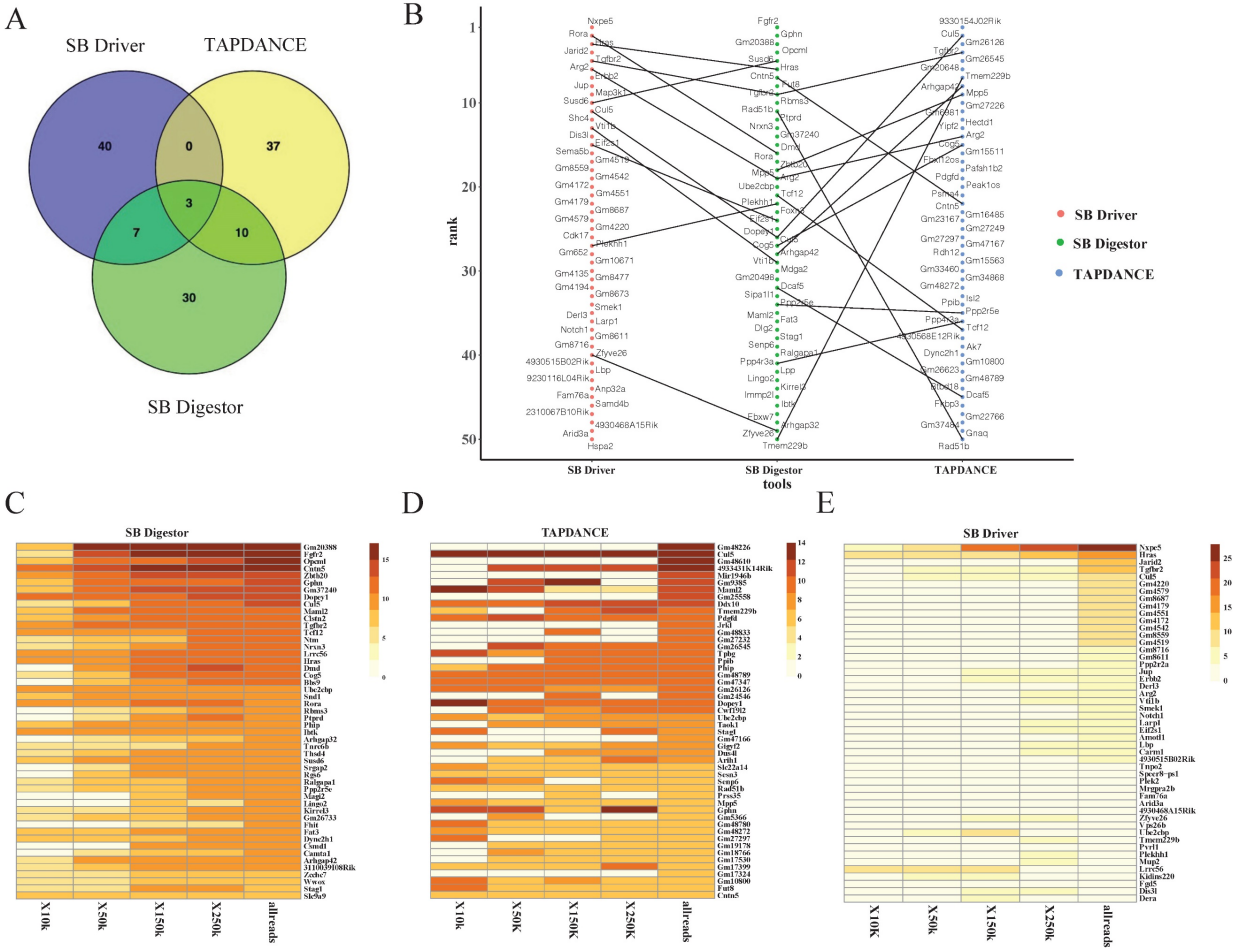
** Comparison of SB Digestor and other tools**. **A-E**. Comparison of the top 50 candidate genes identified with three different tools by Venn diagram (A) and scatter plot (B). To further demonstrate the performance stability of each tool, different numbers of reads were extracted randomly for candidate gene calling with different tools, namely, SB Digestor (C), TAPDANCE (D), and SB Driver (E). Then, the top genes were listed in the heatmap. The color indicates the abundance of each gene in tumor samples.

We next conducted a comparative analysis of our SB Digestor with two previously reported representative SB cancer gene identification algorithms: the gene-centric method and the locus-centric method. For these two algorithms, we selected a typical tool respectively: TA Poisson distribution statistics (TAPDANCE) 16, 22, which is the most commonly used locus-centric tool, and SB Driver 17, the most recent development gene-centric based tool. For TAPDANCE, default parameters were used; for SB Driver, we used the Trunk Driver analysis model, and we set the minimum read depth cutoff as 20. With 67 tumor samples, SB Digestor identified 222 common insertion genes, while TAPDANCE and SB Driver detected 278 and 243 genes, respectively. Then, we compared the top 50 candidate genes generated from these three tools. The Venn diagram showed that the overlap among these three tools tended to be low, with only 3 genes. SB Digestor shares 13 and 10 genes with TAPDANCE and SB Driver, respectively (Fig. 4A). As shown in Fig. 4B, cross-comparison of these genes from each approach, SB Digestor provides the most representative candidates in both rank and gene number, indicating SB Digestor could cover the majority of the top genes identified by other tools.

In addition, to evaluate how stably SB Digestor performed with the different number of sequenced reads, for the above test sample cohort, we chose 33 samples with sequenced reads numbers more than 250000, then we randomly extracted 10000, 50000, 150000, and 250000 reads for each sample to conduct common insertion gene identification by using SB Digestor, TAPDANCE, and SB Driver.

As shown in Fig. 4C, the top genes were consistently identified by SB Digestor regardless of the number of reads extracted, while the other two algorithms did not yield consistent results (Fig. 4D, E). This indicates that after taking tumor heterogeneity into account, the algorithm of SB Digestor is more suitable for identifying SB tumor driver genes, even at a lower sequenced read number. Thus, when compared to the uniform depth cutoff for all SB tumors used by SB Driver and TAPDANCE, the depth cutoff tailored for each sample is more appropriate. From a biological perspective, due to tumor heterogeneity, the driver genes for each tumor or even the different cells from one tumor are different. Additionally, although more sequenced reads indeed enable the detection of more driver genes, SB Digestor can compensate even when a sample has fewer read numbers.

### SB Digestor more accurately identifies functional drivers

**Figure 5:**
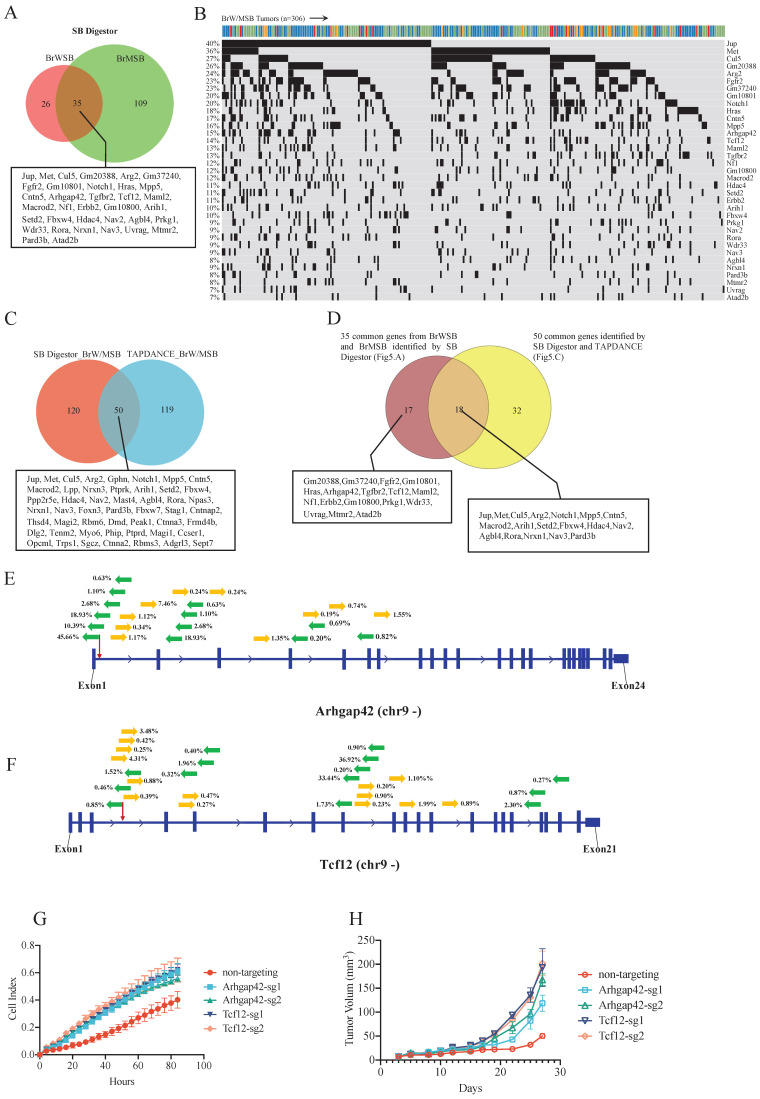
** Candidate gene validation**. **A.** Venn diagram indicating CIS genes for the BrWSB and BrMSB groups by using SB Digestor. **B.** Oncoplot shows the top overlapping 35 genes in both BrWSB and BrMSB tumors and their frequency in all tumor samples. **C.** Venn diagram showing the candidate genes identified by SB Digestor and previously by TAPDANCE. **D.** Venn diagram showing 18 overlapping genes among the 35 common genes identified by SB Digestor (Fig. 5A) and 50 common genes (Fig. 5C). **E-F.** SB transposon insertion patterns (appearing at more than 0.2%) in Arhgap42 and Tcf12. **G.** Candidate tumor suppressor genes were knocked out by using the CRISPR‒Cas9 system in G600 cells to evaluate their function. Cell proliferation was monitored with real-time cell analysis. **H.** Candidate gene knockout tumor cells and control cells were inoculated into nude mice for tumorigenesis evaluation.

In our previous study 5, to identify genes involved in Brca1-related tumorigenesis, we collected 306 tumors from 4 transgenic mice strains: Brca1^Co/Co^; WAP-Cre; SB; T2Onc3-12740 (BrWSB40), Brca1^Co/Co^; WAP-Cre; SB; T2Onc3-12775 (BrWSB75), Brca1^Co/Co^; MMTV-Cre; SB; T2Onc3-12740 (BrMSB40), and Brca1^Co/Co^; MMTV-Cre; SB; T2Onc3-12775 (BrMSB75) 5. Using the same cutoff criteria, that is, appearance in at least 5% of tumors, in the 12740 and 12775 strains, SB Digestor identified 61 putative driver genes (Supplementary Table 2) from the BrWSB group and 144 genes from the BrMSB group (Supplementary Table 3). Combining the two groups yielded a total of 170 (Supplementary Table 4) distinct genes, including 35 genes that were mutated by SB in both BrWSB and BrMSB mice (Fig. 5A). These 35 genes were mutated at frequencies ranging from 7-40% and should be considered the top tier of candidates (Fig. 5B). Next, we compared the 170 newly identified genes with the 169 driver genes previously identified by TAPDANCE 5. The data revealed that 50 genes were common, with 120 and 119 genes that were specifically identified by SB Digestor and TAPDANCE, respectively (Fig. 5C). Further comparison between this 50-gene list and the 35-gene list identified 18 common genes, whereas 32 and 17 genes appeared in the separate lists (Fig. 5D).

Among the 17 genes that were not identified by TAPDANCE, there were 3 groups of genes. The first group of genes was well-known cancer-related genes, i.e., Fgfr2, Hras, Tgfbr2, Nf1, and Erbb2. The second group contains genes that were originally annotated by MGI but were not officially named yet (Gm20388, Gm37240, Gm10801, and Gm10800). The third group of genes includes Arhgap42, Tcf12, Maml2, Prkg1, Wdr33, Uvrag, Mtmr2, and Atad2. Some of these genes have been implicated in cancer, although their functions remain unclear. Next, we conducted functional validation of two genes, Arhgap42 and Tcf12, which were mutated by SB at 15% and 14%, respectively (Fig. 5B). The insertion patterns and directions were distributed randomly for both genes (Fig. 5E, F), indicating that they are tumor suppressors. To provide further evidence for this model, we disrupted these two genes in G600 cancer cells by CRISPR‒Cas9-mediated gene editing. Our data indicated that knocking out these genes could significantly increase cell proliferation *in vitro* (Fig. 5G). Furthermore, implantation of G600 cells into the fat pad of nude mice demonstrated that loss of Arhgap42 or Tcf12 dramatically increased tumorigenesis (Fig. 5H).

### Oncogenic functions of Fgf/Fgfr family members in the Brca1-associated tumorigenesis revealed by SB Digestor

**Figure 6:**
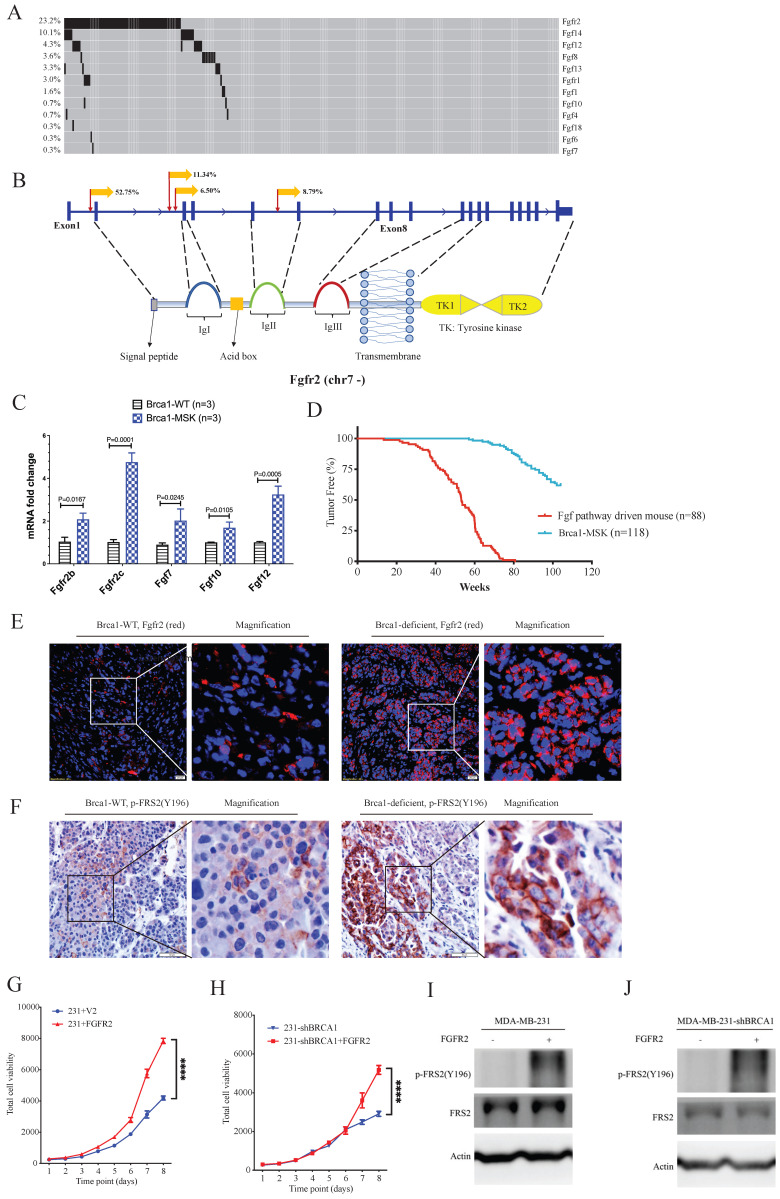
**The Fgf/Fgfr pathway is a potent gain-of-function pathway for tumorigenesis. A.** Oncoplot of the Fgf/Fgfr-related genes in both BrWSB and BrMSB tumors showing their frequency in all tumor samples. **B.** Representation of the distribution (percentage more than 5%) of CISs in the gene Fgfr2. Predicted effect of candidate genes, as indicated by their sense fraction of insertions based on the direction of the CAG promoter and the transcriptional direction of the inserted gene. **C.** The qPCR data revealed the Fgfs (Fgf7, Fgf10, Fgf12) and Fgfr2 (Fgfr2b, Fgfr2c) mRNA levels in Brca1 wild-type and deficient tumors (n=3). **D.** Kaplan-Meier curve showing the mammary tumor-free rate for SB mice with Fgfs/Fgfr-driven mice (n = 88) and control mice (n=118): BrW (n = 62) and BrM (n = 56). Fgf/Fgfr-related tumors tended to show earlier onset than control tumors (p < 0.0001) according to the log-rank test. **E-F.** IF/IHC staining shows the comparison of Fgfr2 expression (E) and Fgfr2 downstream phosphorylation (F) levels between Brca1 wild-type and Brca1-deficient mouse tumors. **G.** Cell viability comparison between control and Fgfr2-activated MDA-MB-231 cells. **H.** Brca1 knockdown and Brca1 knockdown with Fgfr2 activation in MDA-MB-231 cell lines. **I. J.** Representative Western blot showing Fgfr2 activation and Brca1 knockdown with Fgfr2 activation in the MDA-MB-231 cell line.

Next, we focused on fibroblast growth factor receptor 2 (Fgfr2), which has been implicated in the breast cancer formation 23-26. The SB Digestor identified SB-mediated mutations in Fgfr2 in 23% of tumors (Fig. 6A), whereas it was not identified by the other two approaches. To further evaluate whether the gene Fgfr2 plays an active role in tumorigenesis, we first studied the insertion patterns of the SB transposon. Among 628330 reads, 551166 (87.7%) demonstrated transposon inserts that engage the CAG promoter in the same manner as the Fgfr2 transcript (Fig. 6B), suggesting that the transcription of Fgfr2 might be upregulated in these tumors. Consistently, real-time RT‒PCR analysis detected higher mRNA expression levels of Fgfr2 in Brca1 mutant cells (Fig. 6C), suggesting that Fgfr2 may act as an oncogene downstream of Brca1 to enhance Brca1-associated tumorigenesis. Further on this line, we have recently demonstrated that mammary activation of Fgfr2 signaling in transgenic mice could initiate mammary tumorigenesis by suppressing Brca1 via the ERK-YY1 axis 27. Thus, these results not only highlight the power of SB Digestor in the identification of cancer drivers, but also reveal a potential reciprocal regulation of Brca1 and Fgfr2 signaling during tumorigenesis, which certainly deserves further studies.

Fgfr2 is one of the four membrane-spanning tyrosine kinases that mediate the signaling of at least 22 fibroblast growth factors (Fgfs) 28. Next, we analyzed the involvement of other Fgfr and Fgf family members. The data revealed 100 tumors in 88 mice carrying SB insertions in various members of the Fgf/Fgfr families (Fig. 6A). RT‒PCR analysis also detected increased expression of Fgf7, Fgf10, and Fgf12 (Fig. 6C). Comparison of tumorigenesis in these 88 mice with that in the control mice, which carry only mammary-specific Brca1 knockout by MMTV-Cre or WAP-Cre (Brca1-MSK n=118), indicating that the mice with Fgf pathway activation exhibited much faster tumor progression than the control mice (Fig. 6D). The protein levels of Fgfr2 and FRS2 (an Fgfr2 direct downstream gene) were also higher in Brca1-MSK/SB tumors than in Brca1-MSK tumors (Fig. 6E, F). These data suggest that activation of the Fgf pathway is a potent driver of Brca1-associated tumorigenesis. To further validate its function during tumor progression, we overexpressed Fgfr2 in MDA-MB-231 (231) cells (Fig. 6G) and 231-shBrca1 cells (Fig. 6H), and the results showed that overexpression of Fgfr2 could dramatically activate tumor cell growth and downstream targets, regardless of whether Brca1 was wild-type (Fig. 6G, I) or knocked down (Fig. 6H, J).

Taken together, these data indicated that our newly developed software, SB Digestor, could identify cancer drivers much more efficiently and consistently regardless of read number and tumor heterogeneity.

## Discussion

High-throughput sequencing-based technology, including whole genome/exome analysis and transcriptomic analysis, has helped to illustrate the genomic landscape of human cancers 29, 30. However, it is still challenging to distinguish drivers based only on the analysis of massive genomic resources. Due to Sleeping Beauty transposon's inherent ability to continually move among chromosomal locations, this transposon mutagenesis system offers a function-based approach to precisely identify driver genes to reveal how cancer develops and evolves 2, 31, 32. Also, it can spontaneously and continually modulate driver genes in an unbiased manner in an *in vivo* tumor model, it is perfectly adapted to various experiments that could help researchers decipher the impact of the tumor microenvironment on the cancer biology 33, 34. Therefore, it is critical to comprehensively decipher the SB insertional spectrum.

Previous studies have applied different tools and algorithms to characterize the SB insertion spectrum to identify SB-trapped driver genes 16, 17, 19. The locus-centric algorithms, such as TAPDANCE and GKC, can effectively identify highly condensed SB insertion sites, however, they are moderately effective in defining scattered SB insertions, and therefore, the insertion sites identified are fewer and relatively concentrated. Although the most recently developed gene-centric tool SB Driver identifies driver genes in an unbiased manner, which can complement some scattered SB insertion genes, the sequence depth cutoff determination of this software is experience based and the same value for all tumors, which would compromise its ability to eliminate artificial effects and correctly account for the intertumoral heterogeneity. The intertumoral heterogeneity might cause the failure of driver gene identification. For example, if we have 3 tumors with the same number of sequenced reads, 10000, due to tumor heterogeneity, if the driver gene numbers of these 3 tumors are 10, 100, 1000, then the average depths of these genes are 1000, 100, 10. Therefore, if we set 1000 as the depth cutoff, we would miss many true-positive candidate genes in the last two samples, but if we set it as 10, we may obtain many false-positive candidate genes in the first two samples.

In this study, we have developed SB Digestor, which is a gene-centric and user-friendly, Perl-coded tool to enhance tumor driver gene identification and make better use of the SB transposon system. In our computational algorithm, we consider not only statistical significance, similar to other tools, but also the biological aspect-tumor heterogeneity. We first detect significant SB insertion genes by binomial test. Then we conducted saturation analysis to describe the relationship between identified gene number and sequenced read number for each tumor individually to identify the intertumoral heterogeneity, based on which we calculated the tailored driver gene identification parameters (constant a and b) for further data processing. As each tumor was analyzed separately, we obtained stable results, no matter how many sequence reads were available or how large-scale the samples were. The application of saturation analysis enables a more exact correlation between the read number and identified gene number. Therefore, it benefits the data depth cutoff threshold determination. The principle might also be adapted for general tumor genome analysis.

As we expected, when we reanalyzed our previously published function-based driver gene screening dataset (306 tumors) 5, we identified 170 driver genes responsible for Brca1-related tumorigenesis. Among these, we have identified additional well-known drivers, including Erbb2 and Hras, and other potential drivers, such as Fgfr2, Arhgap42, and Tcf12, which failed to be discovered by other tools. In our 67 training samples, compared to other tools, SB Digestor showed more stable performance with different numbers of randomly extracted sequencing reads, which indicates that even a driver clone at an initial stage or present at a lower proportion could also be detected by our algorithm. This study also provided some clues to explain why some important tumor driver genes, such as Fgfr2, were missed in our previous study 5. Further validation of Arhgap42 and Tcf12, the functions of which in cancer were previously unclear, indicated that both serve as tumor suppressor genes for Brca1-associated tumorigenesis.

Many previous studies have identified Fgfr2 as an oncogene for breast cancer 35, 36, yet its role in BRCA1-associated breast cancer remains unclear. Using both parental and shBrca1 knockdown MDA-MB-231 cells, we demonstrated that overexpression of FGFR2 could dramatically promote tumor cell growth and activate downstream targets, illustrating the oncogenic role of FGFR2 and its related signaling in breast cancers regardless of BRCA1 status. To further illustrate the role of Fgfr2 in BRCA1-associated breast cancer, we studied a mouse strain carrying an Fgfr2 allele (*Fgfr2^pLoxpneo-S252W^*) 37 that can be activated specifically in mammary tissue after crossing with a Cre transgenic mouse 38. We demonstrated that the activation of Fgfr2 signaling could initiate tumor formation by suppressing Brca1 via the ERK-YY1 axis. Our subsequent functional study in Fgfr2/Brca1 double mutant mice confirmed the cooperation between Fgfr2 activation and Brca1 deficiency in accelerating mammary tumorigenesis 27. Thus, these results indicate the accuracy of SB Digestor in the identification of cancer drivers.

In summary, after considering both statistical and biological factors in our computational algorithm, the performance of SB Digestor is enhanced greatly in terms of both accuracy and stability. SB Digestor could identify the intertumoral heterogeneity and shape the relationship between sequenced read number and identified driver gene number. Based on this property, it defines an appropriate read depth cutoff for each tumor to dissect the effects of tumor heterogeneity to further help driver gene detection. More specifically, it can avoid the obvious drawbacks of using a uniform depth threshold for all tumors, the main manifestations of which are the exclusion of true driver events when the uniform depth is too high and the inclusion of false-positive events when it is too low. Thus, the tool we have provided, SB Digestor, can enhance the utility of SB insertional mutagenesis to prioritize drivers and enhance our understanding and interrogation of the natural course of cancers.

## Materials and Methods

### DNA sequencing and data pre-processing

SB mouse experiments were performed as in our previously published paper 5. Sixty-seven tumor samples from Sleeping Beauty transposon screens were collected. DNA sequences with transposon were identified and enriched by restriction enzymes BfaI and NlaIII. Then a sequence library was prepared by using Splinkerette-PCR 39. Following that is a second round of PCR with SB Illumina adaptors. DNA sequencing was performed with 150 bp paired-end reads on the Illumina HiSeq X Ten platform. The sequence reads were then filtered by removing sequencing adapters, SB transposon sequences and splinkerette linker sequences by using cutadapt version 1.18. Here, we also discarded the processed sequences that were shorter than 20 bp since they were prone to mapping to multiple genomic locations. Then we obtained the clean reads. The clean reads were aligned to the mouse reference genome (mm10: Mus_musculus.GRCm38.dna.primary_assembly.fa) by bowtie2 version 2.2.5 with default parameter.

The genome was annotated with genes by using Mus_musculus.GRCm38.94.gff3 (n=54532 genes, including bidirectional_promoter_lncRNAs, ncRNAs, pseudogenes, and genes). Here, we aimed to identify either protein-coding genes or other noncoding genome structures that contribute to tumorigenesis. Note that the GFF3 file was downloaded from Ensembl. Other annotation sources, such as UCSC and GENCODE, could also be used.

### Gene knockout and functional validation

Candidate genes were knocked out by using the CRISPR‒Cas9 system with sgRNA, Arhgap42-sg1 (AGTCACTGAAAGAATTCGCA), Arhgap42-sg2 (GACTTCCAGTTTGAGTGTAT), Tcf12-sg1 (AGTAGTCAGTTCAGCGGGTC) and Tcf12-sg2 (ACTTACTCTAGATGAATCAT) or overexpressed with pBp-FGFR2c-WT in the G600 cell lines (Addgene plasmid No. 45699).

Cell growth curves were measured according to the cellular density at seeding using impedance measurements with the xCELLigence Real-Time Cell Analysis system (Agilent Technologies) with an E-plate.

All mouse experiments were performed under the ethical guidelines of the University of Macau (animal protocol number: UMAEC-037-2015). Mice were housed in a specific-pathogen-free (SPF) facility at 23-25 °C on a 12-h light/dark cycle. Cultured G600 cells were dissociated into single cells and resuspended in 50% Matrigel (Corning, 356234) for inoculation. Nude mice were anesthetized with tribromoethanol, and a small abdominal incision was made. Mammary fat pads were exposed gently by forceps, and 1 million cells were injected using a microliter syringe with a 27-gauge needle. Tumor volume was calculated as V = (W^2^ × L)/2.

Quantitative Real-Time (qRT)-PCR: A QuantiTect reverse transcription kit (205313; Qiagen, Hilden, Germany) was used for reverse transcription, and RT‒qPCR was performed by a QuantStudio 7 Flex real-time PCR system (Thermo Fisher Scientific, Waltham, MA). The primer sequences are listed in Table 1.

Immunofluorescence (IF) & Immunohistochemistry (IHC) staining: Tumor tissue sections were fixed with paraformaldehyde (4% v/v). Deparaffinized thin sections of the tumors were heated with Retriever (62700-10; Electron Microscopy Sciences, Hatfield, PA) in Buffer A (citrate; pH 6.0) followed by antibody staining and Fgfr2 (Abcam, ab10648, 1:500) and p-FRS2 (Abcam, ab10425, 1:500) antibodies. A NikonA1R confocal system (NikonCorp., Tokyo, Japan) was used to acquire images.

Viral Infection and Western blotting: Lentivirus were used to infect MDA-MB-231 or MDA-MB-231-shBRCA1 cells with FGFR2, and infected cells were selected; then, these selected cells were used for cell viability or Western blotting assays. Western blotting antibodies were as follows: anti-FGFR2 (Abcam, ab10648, 1:1000); phospho-FRS2-α (CST, #3864L, 1:1000); and β-actin (A5316, Sigma, 1:4000).

## Supplementary Material

Supplementary tables.

## Figures and Tables

**Table 1:** Primer sequences

Oligonucleotides	
Primer: mFgfr2 IIIb	Forward: AAGGTTTACAGCGATGCCCA
Primer: mFgfr2 IIIb	Reverse: AGAGCCAGCACTTCTGCATT
Primer: mFgfr2 IIIc	Forward: GTGTTAACACCACGGACAAA
Primer: mFgfr2 IIIc	Reverse: TGGCAGAACTGTCAACCATG
Primer: mFgf7	Forward: GAACAAAAGTCAAGGAGCAACC
Primer: mFgf7	Reverse: GTCATGGGCCTCCTCCTATT
Primer: mFgf10	Forward: GAGAAGAACGGCAAGGTCAG
Primer: mFgf10	Reverse: CTCTCCTGGGAGCTCCTTTT
Primer: mFgf12	Forward: GTACCATTGATGGGACCAAGG
Primer: mFgf12	Reverse: ACGCAGTCCTACAGGAATTAGAT

## Abbreviations

SB: Sleeping Beauty
CIS: Common insertion sites
